# Supplementary material for: Ubiquinol-mediated suppression of mitochondria-associated ferroptosis is a targetable function of lactate dehydrogenase B in cancer
Source: Nat Commun. 2025 Mar 16;16:2597. doi: 10.1038/s41467-025-57906-3 (PMC11911438; doi:10.1038/s41467-025-57906-3)
Supplement: Supplementary file 1 — Supplementary Information [file 41467_2025_57906_MOESM1_ESM.pdf]

Supplementary Figure 1

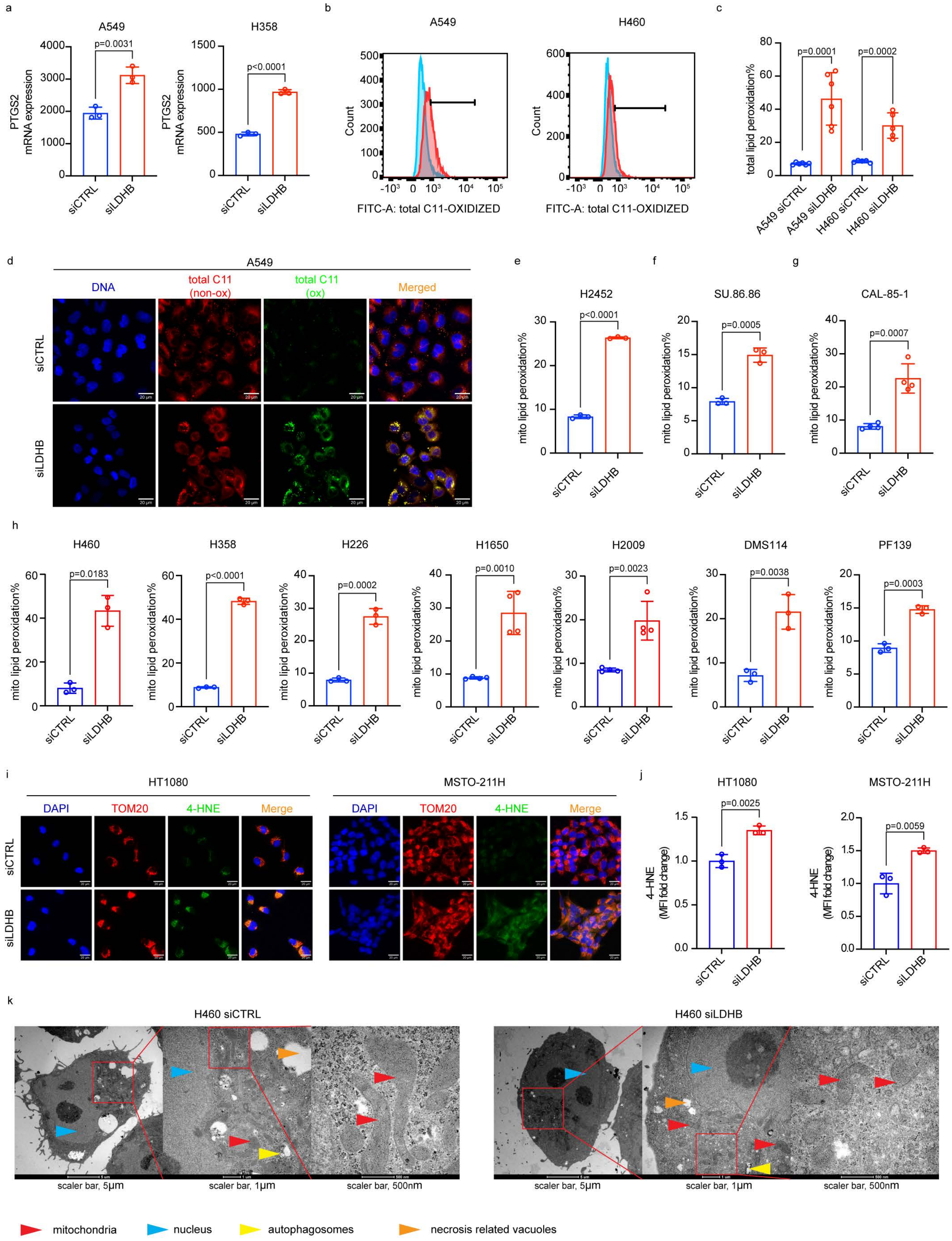

Supplementary Figure 1

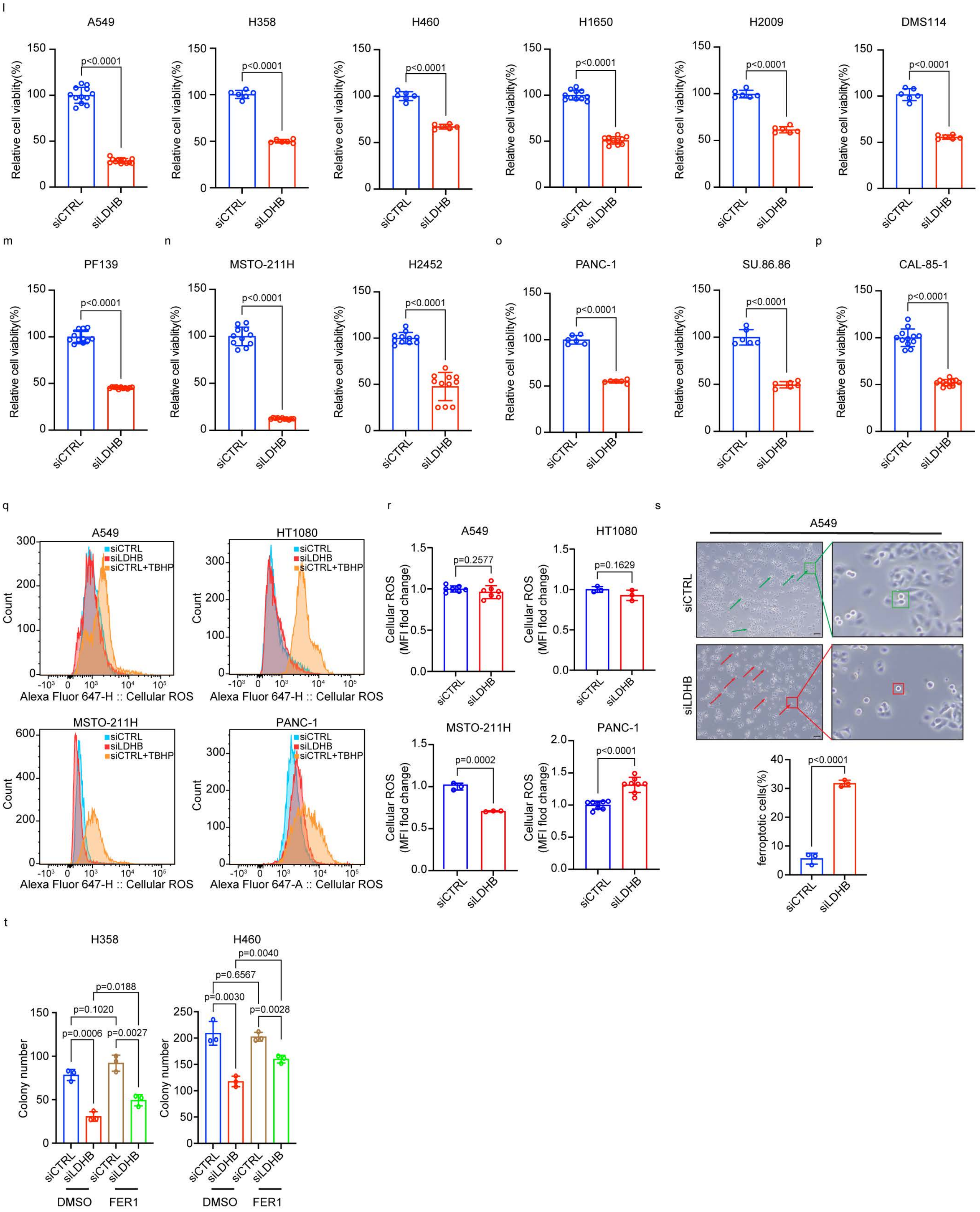

Supplementary Fig. 1 Silencing of LDHB induces mitochondrial lipid peroxidation in cancer cells. mRNA expression of PTGS2 in A549, H358 siRNAs cells, n=3 independent replicates (**a**). Measurement of total lipid peroxidation in A549, H460 siRNAs after 72 h of transfection, n=6 (A549), or 5 (H460) independent replicates (**b, c**). Total lipid peroxidation assessed by immunofluorescence in A549 siRNAs cells after 72 h of transfection (**d,** scale bar, 20μm). Mitochondrial lipid peroxidation assessed in malignant pleural mesothelioma (**e**), pancreatic cancer (**f**), breast cancer (**g**), lung cancer (**h**) siRNAs cells after 72 h of transfection. n=3-4 independent replicates. Colocalization analysis by immunofluorescence in HT1080, MSTO-211H siRNAs after 72 h of transfection **(i, j,** scale bar, 20μm). Transmission electron microscopy images of H460 siRNAs cells after 72 h of transfection (**k**). Analysis of cell viability of siRNAs cells after normalization to siCTRL group, n=3 independent replicates (**l-p**). Cellular ROS analysis in A549, HT1080, MSTO-211H, PANC-1 cells after 72 h of transfection with siRNAs and 100 μM TBHP (tert-butyl-hydroperoxide) treated samples as positive control, n=3-8 independent replicates (**q, r**). Ferroptotic cells (in green and red box) shown by bright field microscope images of A549 siRNAs and counted by Fiji (**s,** scale bar, 20μm). Quantitative analysis of colony numbers of H358, H460 siCTRL and siLDHB cells at day 10 after treatment with DMSO or 2 μM ferrostatin-1 (FER1) for three days after 24 h of transfection, n=3-6 independent replicates (**t**). Data are presented as mean ± SD. Unpaired, two-tailed t test. *ns* no significant difference, *$P < 0.05$, **$P < 0.01$, ***$P < 0.001$, ****$P < 0.0001$. Source data are provided as a Source Data file.

Supplementary Figure 2

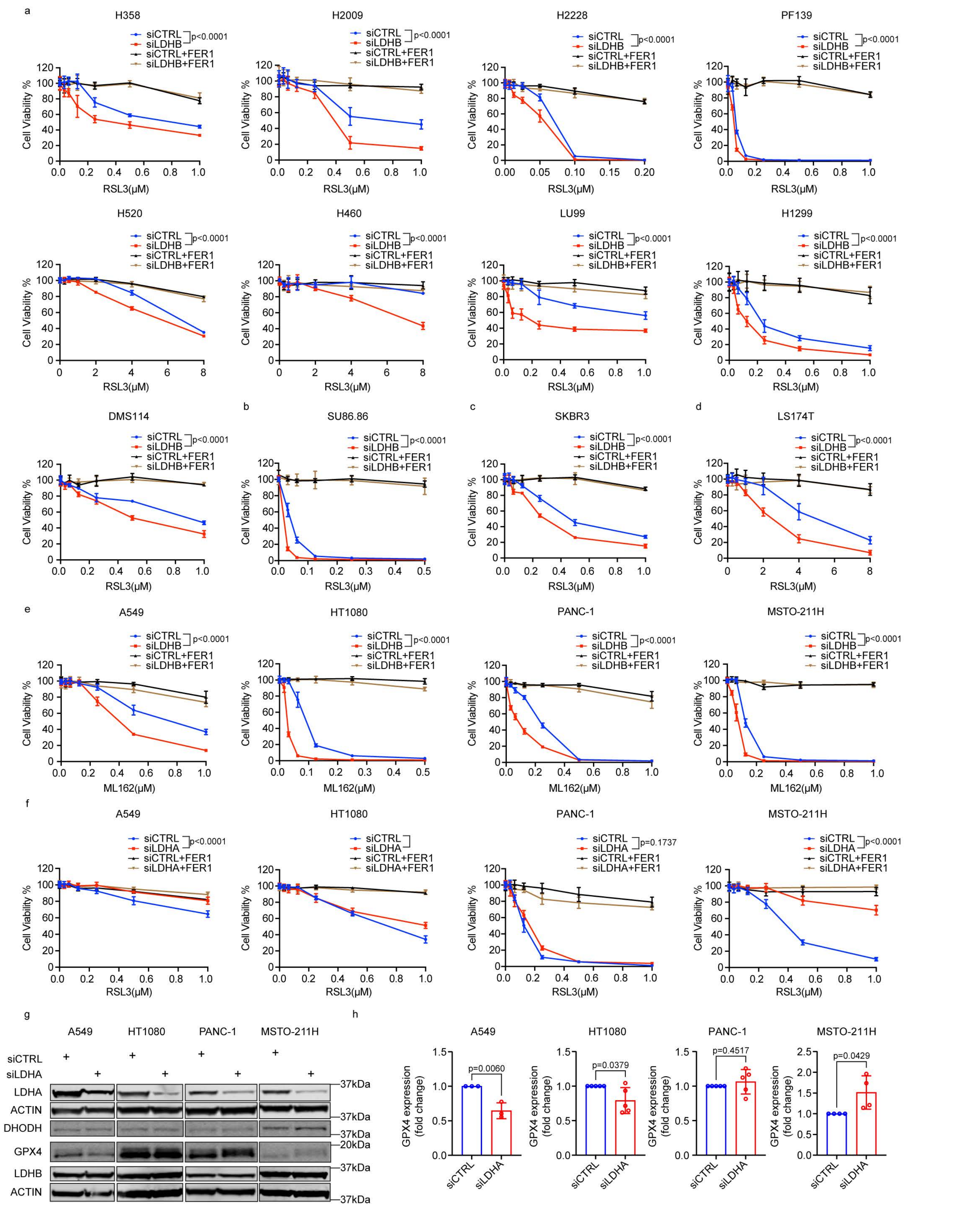

Supplementary Figure 2

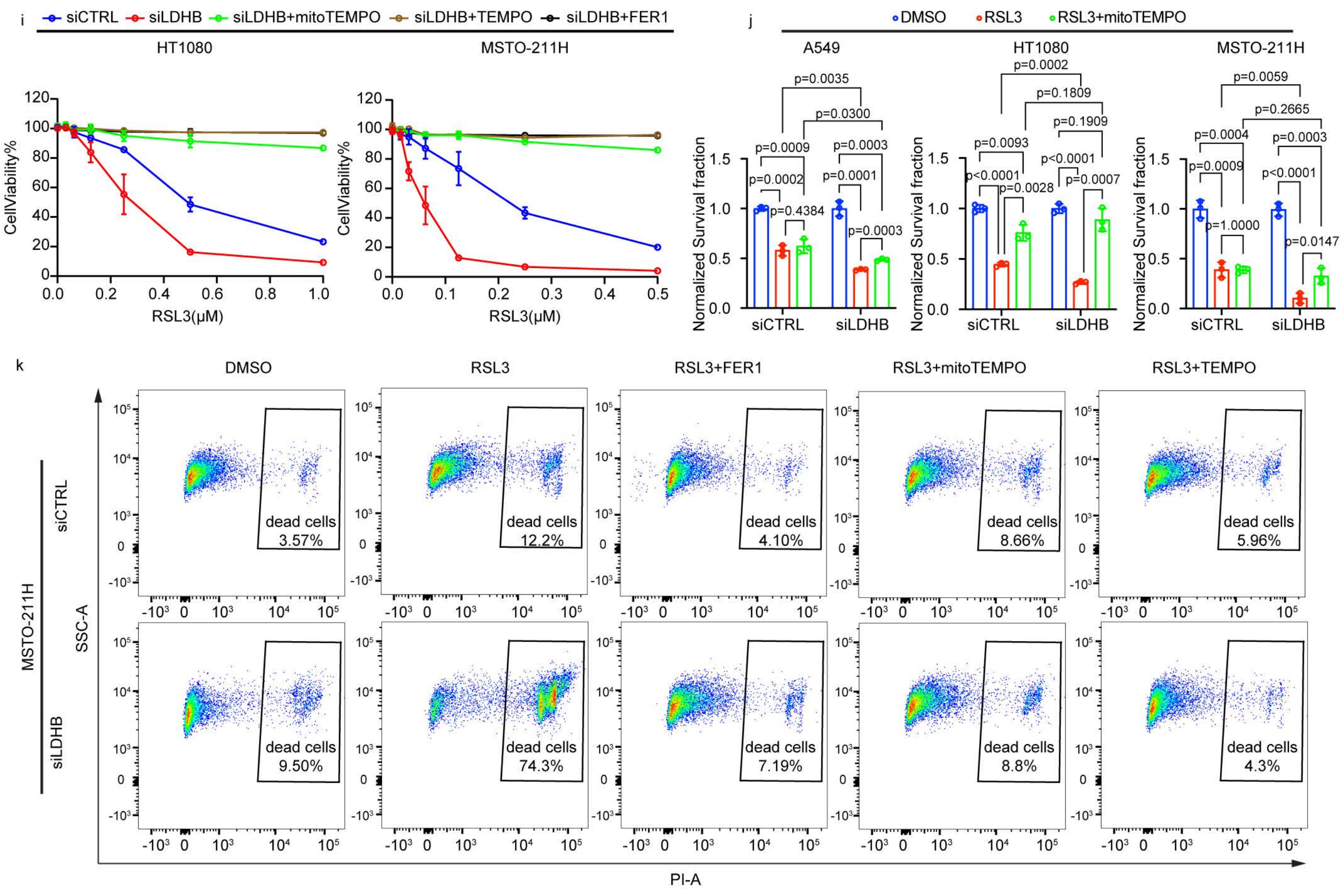

Supplementary Fig. 2 LDHB suppresses mitochondria-associated ferroptosis in cancer cells. Cell viability of siRNAs cells treated with DMSO or GPX4 inhibitors (RSL3 or ML162) alone or in combination with 5 µM FER1 for 48 h, following pretreatment with 5 µM FER1 for 24 h, n=3 (**a, b, d, e**), or 4 independent replicates (**c**). Western blot analysis of the expression of LDHA, GPX4, DHODH, LDHB, and the statistical analysis for GPX4 expression after normalizing to corresponding control in A549 (n=3), HT1080 (n=5), PANC-1 (n=5), MSTO-211H (n=4) siRNAs cells, n indicates independent replicates (**g, h**). Assessment of cell viability by APH assay in HT1080, MSTO-211H siRNAs cells treated with DMSO or RSL3 alone or in combination with 20 µM mitoTEMPO, 20 µM TEMPO, 5 µM FER1 for 48 h after pretreatment with vehicle, 20 µM mitoTEMPO, 20 µM TEMPO, 5 µM FER1 for 24 h, n=3 independent replicates (**i**). Clonogenic survival assay of A549, HT1080 and MSTO-211H siRNAs cells treated with DMSO or RLS3 alone (at the same dose as in Fig.2e) or in combination with 20 µM mitoTEMPO for 48 h after pretreatment with vehicle or 20 µM mitoTEMPO for 24 h and then grown in fresh medium for additional 7 days. The data were normalized to DMSO groups, n=3 independent replicates (**j**). Analysis of PI staining by flow cytometer in MSTO-211H cells after 72 h of transfection with siRNAs treated with DMSO or 250 nM RSL3 alone or in combination with 5 µM FER1, 20 µM mitoTEMPO, 20 µM TEMPO for 24 h. n=3 independent replicates (**k**). Data are presented as mean ± SD. Two-way ANOVA (**a-f**), Unpaired, two-tailed t test (**h, j**). *ns* no significant difference, *$P < 0.05$, **$P < 0.01$, ***$P < 0.001$, ****$P < 0.0001$. Source data are provided as a Source Data file.

Supplementary Figure 3

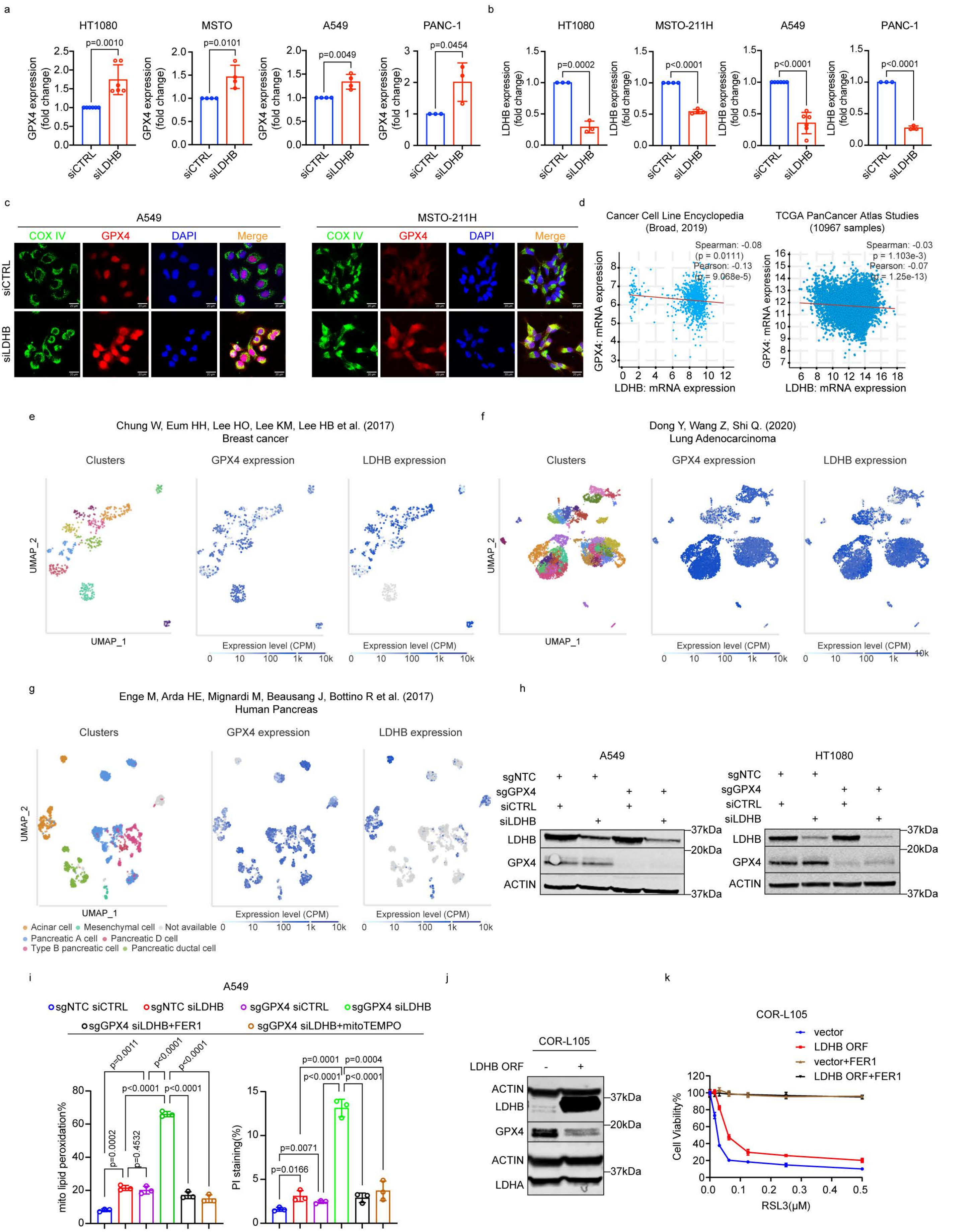

Supplementary Fig. 3 LDHB acts in parallel with GPX4 to suppress mitochondria-associated ferroptosis. Quantitative analysis of GPX4 and LDHB expression in HT1080, MSTO-211H, A549, PANC-1 siRNAs cells by Western blot. (**a, b**). The colocalization of GPX4 and COX IV was investigated by immunofluorescence in A549 and MSTO-211H siRNAs cells after 72 hours of transfection (**c,** scale bar, 20μm). Correlation analysis of mRNA expression of LDHB and GPX4 in 1739 cancer cell lines and 10967 patient samples with cBioPortal (**d**). UMAP plot of single cells from breast cancer, lung adenocarcinoma patients and human pancreas and analysis of GPX4 and LDHB expression with the Single Cell Expression Atlas (**e-g**). Immunoblot analysis of LDHB, GPX4 and ACTIN in A549, HT1080 sgNTC and sgGPX4 cells after 72 h transfection with siRNAs (**h**). Analysis of mitochondrial lipid peroxidation and PI staining in A549 sgNTC, sgDHODH cells after 72 h of transfection with siRNAs, n=3 independent replicates (**i**). Immunoblot analysis of LDHB, GPX4, LDHA and ACTIN in COR-L105 and COR-L105 LDHB ORF cells (**j**). Cell viability assay of COR-L105 cells and COR-L105 LDHB ORF cells treated with RSL3 with or without 5 μM FER1 for 48 h after pretreatment with 5 μM FER1 for 24h, n=3 independent replicates (**k**). Tumor volume (**l**), weight (**m**), representative immunohistochemistry (IHC) images (**n,** scale bar, 50μm) of A549 shCTRL and shLDHB xenograft tumors treated with solvent or RLS3, n=4-8 tumors from different mice. H-score analysis of 4HNE and LDHB, n=6-11 random fields from different tumors (**o)**. Representative IHC images and H-score analysis of A549 (**p, q,** scale bar, 50μm) and HT1080 (**r, s,** scale bar, 50μm) sgNTC shCTRL, sgNTC shLDHB, sgGPX4 shCTRL, sgGPX4 shLDHB xenograft tumors. n=10-12 random fields from different tumors. Data are presented as mean ± SD (**a, b, i, k, m, o, q, s**) or mean ± SEM (**l**). Two-way ANOVA (**l**) or Unpaired, two-tailed t test (**a, b, i, k, o, q, s**). *ns* no significant difference, **P* < 0.05, ***P* < 0.01, ****P* < 0.001, *****P* < 0.0001. Source data are provided as a Source Data file.

Supplementary Figure 4

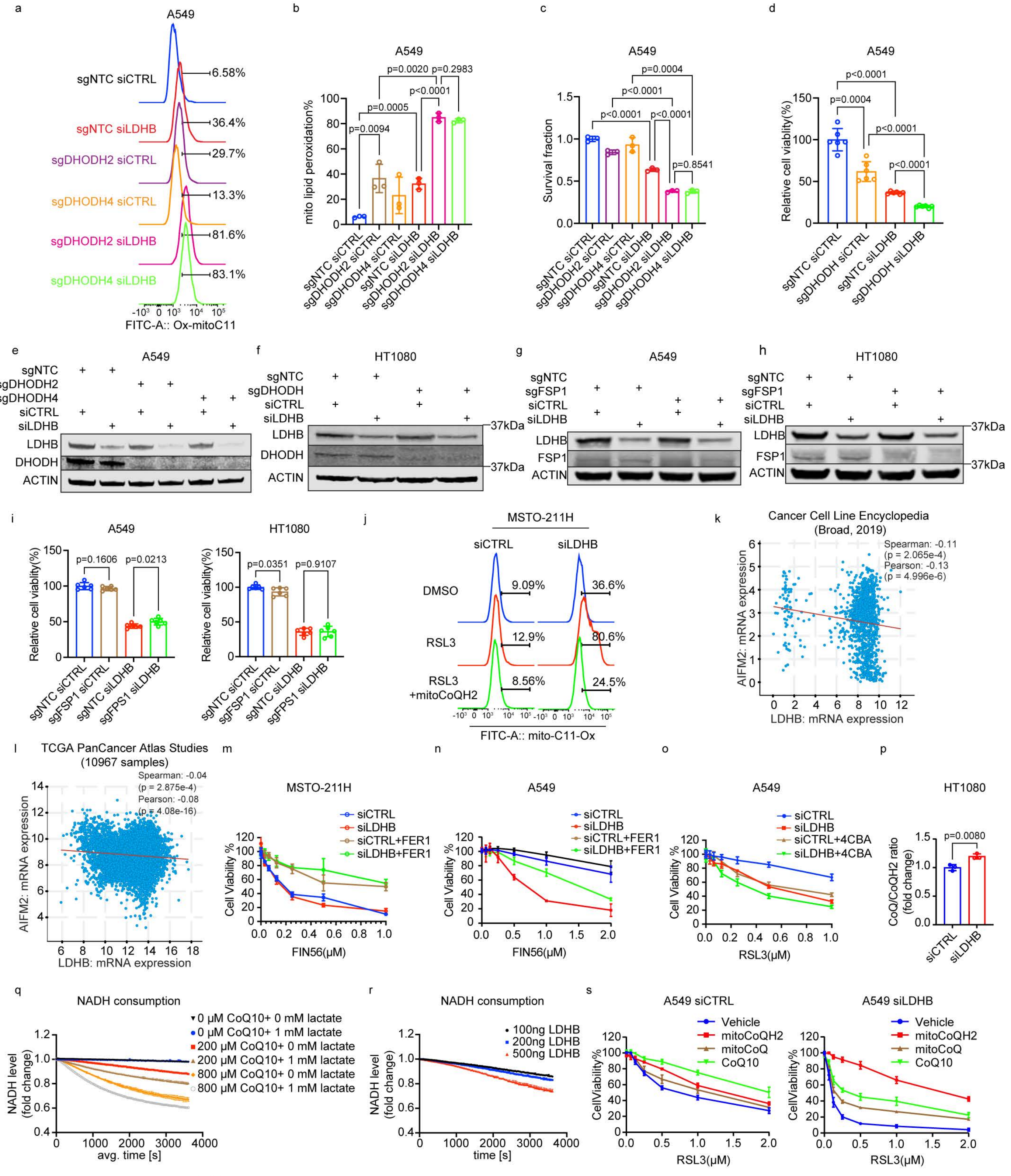

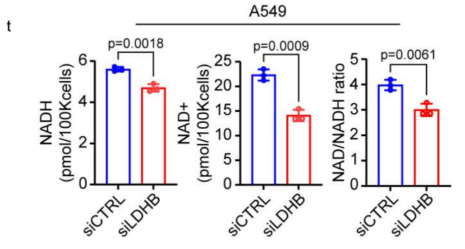

u

Cell line	Pathway name	p value	adj. p value	Mean reaction ratio	#of perturbed genes
A549	Oxidative phosphorylation	2.30E-04	6.24E-04	1.0897	64/100
H358	Oxidative phosphorylation	2.65E-05	1.21E-04	1.1552	52/100

Supplementary Figure 4

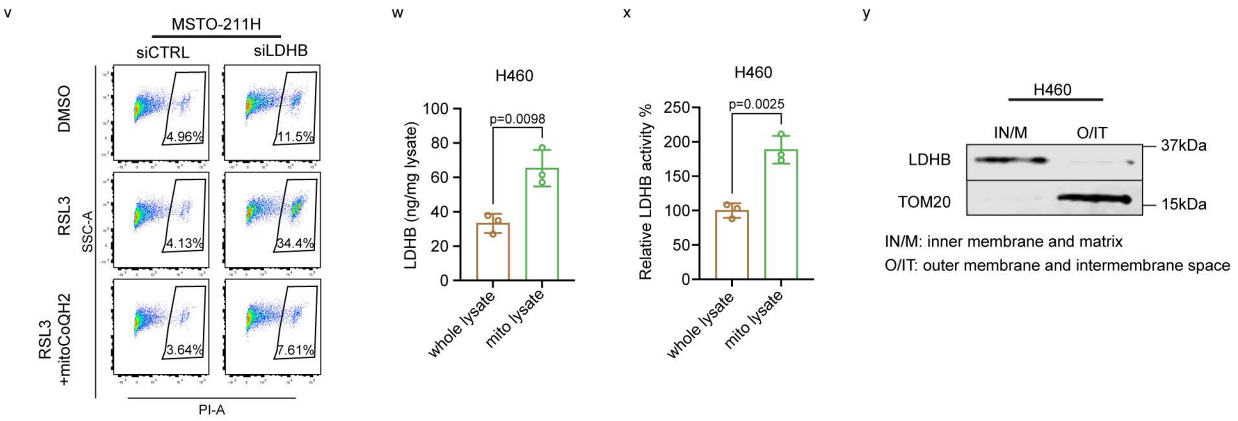

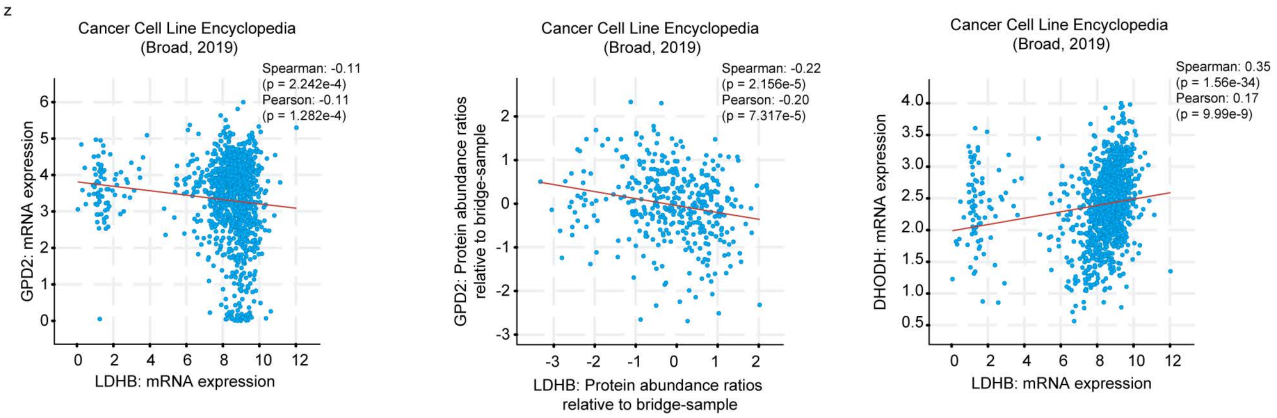

Supplementary Fig. 4 LDHB suppresses ferroptosis by regulating the reduction of CoQH2 in mitochondria. Analysis of mitochondrial lipid peroxidation (**a, b**), survival fraction (**c**), relative cell viability (**d**) in A549 sgNTC and sgDHODH cells after 72 h of transfection with siRNAs. n=3-6 independent replicates. Analysis of the expression of LDHB, DHODH or FSP1 in A549 and HT1080 sgNTC, sgDHODH or sgFSP1 cells after 72 h of transfection with siRNAs (**e-h**). The analysis of relative cell viability in A549 and HT1080 sgNTC, sgFSP1 cells after 72 h of transfection with siRNAs, n=3 independent repeats (including 2 technical repeats of each independent repeat) (**i**). Assessment of mitochondrial lipid peroxidation in MSTO-211H cells after 72 h of transfection with siRNAs treated with DMSO or RSL3 alone or in combination with 500 nM mitoCoQH2 for 1 h (**j**). Correlation analysis of LDHB and AIFM2 in cancer cell lines and patient samples using cBioPortal (**k, l**). Cell viability in siRNAs cells treated with DMSO or FIN56 (**m**, **n**) or RSL3 (**o**) alone or in combination with 5 µM FER1 (**m**, **n**) or 5 mM 4CBA (**o**) for 48 h, following pretreatment with 5 µM FER1 (**m, n**) or 5 mM 4CBA (**o**) for 24 h. n=3 independent replicates. CoQ/CoQH2 ratio in HT1080 siRNAs cells (**p**). NADH consumption assay (A340 nm) in TBS buffer with or without lactate at different concentrations of CoQ10 (**q**), and different LDHB concentration without lactate (**r**). Cell viability of A549 siRNA transfected cells treated with DMSO or RSL3 alone or in combination with 100 nM mitoCoQH2 or 100 nM mitoCoQ or 10 µM CoQ10, for 48 h, following pretreatment with vehicle, 100 nM mitoCoQH2 or 100 nM mitoCoQ or 10 µM CoQ10 for 24 h, n=3 independent replicates (**s**). Measurement of NAD and NADH in A549 cells after 72 hours of transfection with RNAs (**t**). Transcriptomics-driven metabolic pathway analysis (TDPA) of the RNA seq data from A549 and H358 siCTRL and siLDHB cells (**u**). Flow cytometer plot of PI staining in MSTO-211H transfected cells after treatment with 0.75 µM RSL3 for 6 h (**v**). Measurement of LDHB protein (**w**) and activity (**x**) in H460 whole and mitochondrial lysate. Immunoblot analysis of LDHB expression in inner and outer mitochondrial membrane (**y**). Correlation analyses of LDHB, GPD2, DHODH in cancer cell lines using cBioPortal (**z**). Data are presented as mean ± SD. Unpaired, two-tailed t test. *ns* no significant difference, $^{*}P < 0.05$, $^{**}P < 0.01$, $^{***}P < 0.001$, $^{****}P < 0.0001$. Source data are provided as a Source Data file.

Supplementary Figure 5

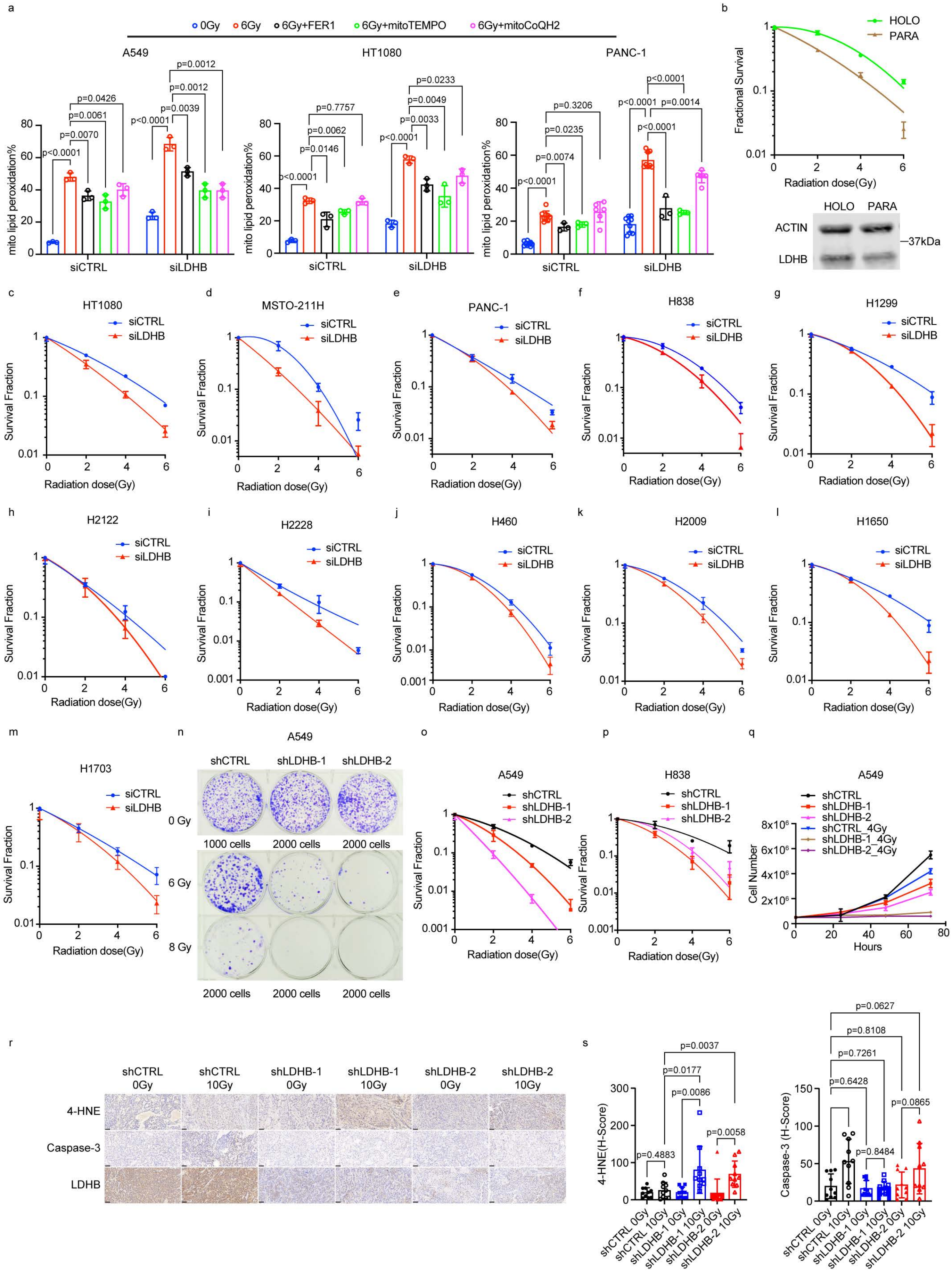

Supplementary Figure 5

t

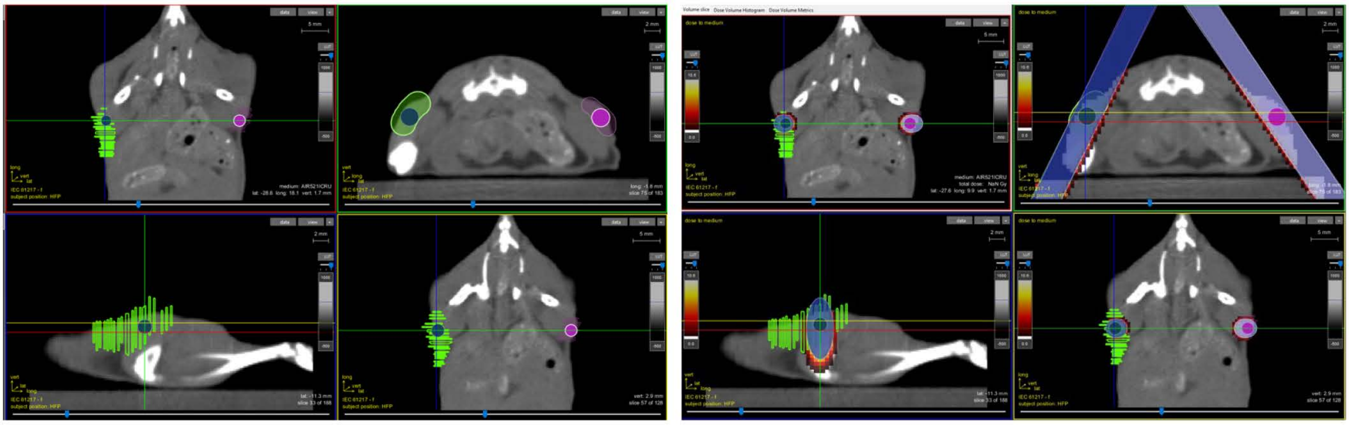

u

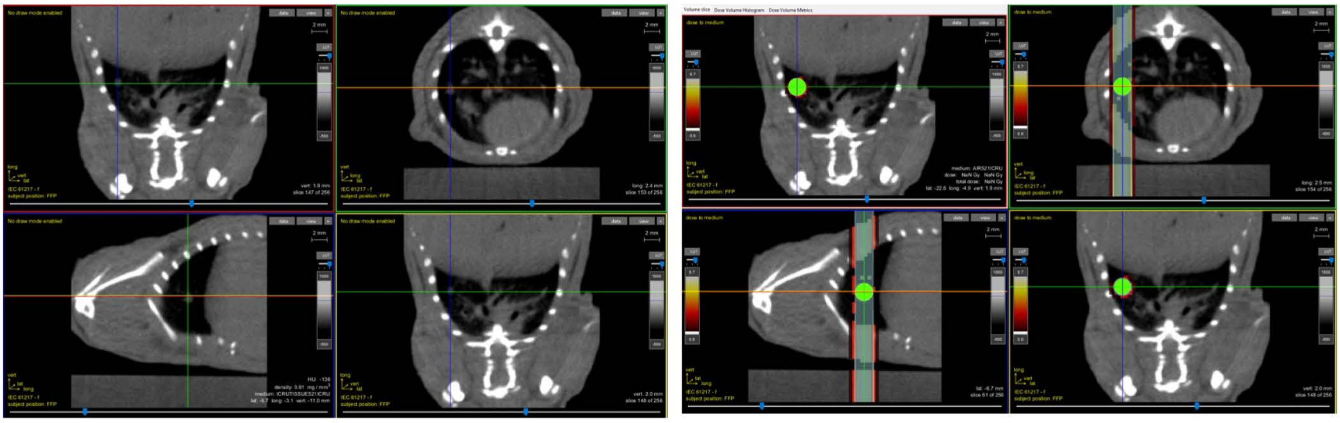

v

Median Survival

Low expression cohort (months)	High expression cohort (months)
41	25

Supplementary Fig. 5 Inhibition of LDHB sensitizes tumor cells to radiotherapy by enhancing mitochondria-associated ferroptosis. Analysis of mitochondrial lipid peroxidation in A549, HT1080, PANC-1 siRNAs cells after 24 h of 6Gy irradiation with or without 5 µM FER1, 20 µM mitoTEMPO, 500 nM mitoCoQH2 treatment. n=3 independent replicates (**a**). Clonogenic survival curve of HOLO and PARA cells after irradiation with doses from 0 to 6 Gy (**b**). Immunoblot analysis of LDHB and ACTIN in HOLO and PARA cells (**b**). Clonogenic survival curve of siCTRL and siLDHB cells after irradiation with doses from 0 to 6 Gy (**c-m**). Images of the clonogenic assay of A549 shCTRL and shLDHB cells irradiated from 0 Gy to 8 Gy (**n**). Clonogenic survival curve of A549 and H838 shCTRL and shLDHB cells after irradiation with doses from 0 to 6 Gy (**o, p**). Cell number of A549 shCTRL and shLDHB cells after exposure to 4 Gy at different time points (**q**). Representative IHC images and H-score analysis of 4-HNE and caspase-3 in A549 shCTRL and shLDHB xenograft tumors after treatment with a single dose of local IR, n=10 random fields from different tumors (**r, s,** scale bar, 50µm). Schematic representation of the plan for local irradiation of xenografts and orthotopic lung tumors (**t, u**). Kaplan-Meier median survival analysis of high and low LDHB groups in patients with lung cancer who received radiotherapy (**v**). Data are presented as mean ± SD. Unpaired, two-tailed t test. *$P < 0.05$, **$P < 0.01$, ***$P < 0.001$, ****$P < 0.0001$. Source data are provided as a Source Data file.

Supplementary Figure 6

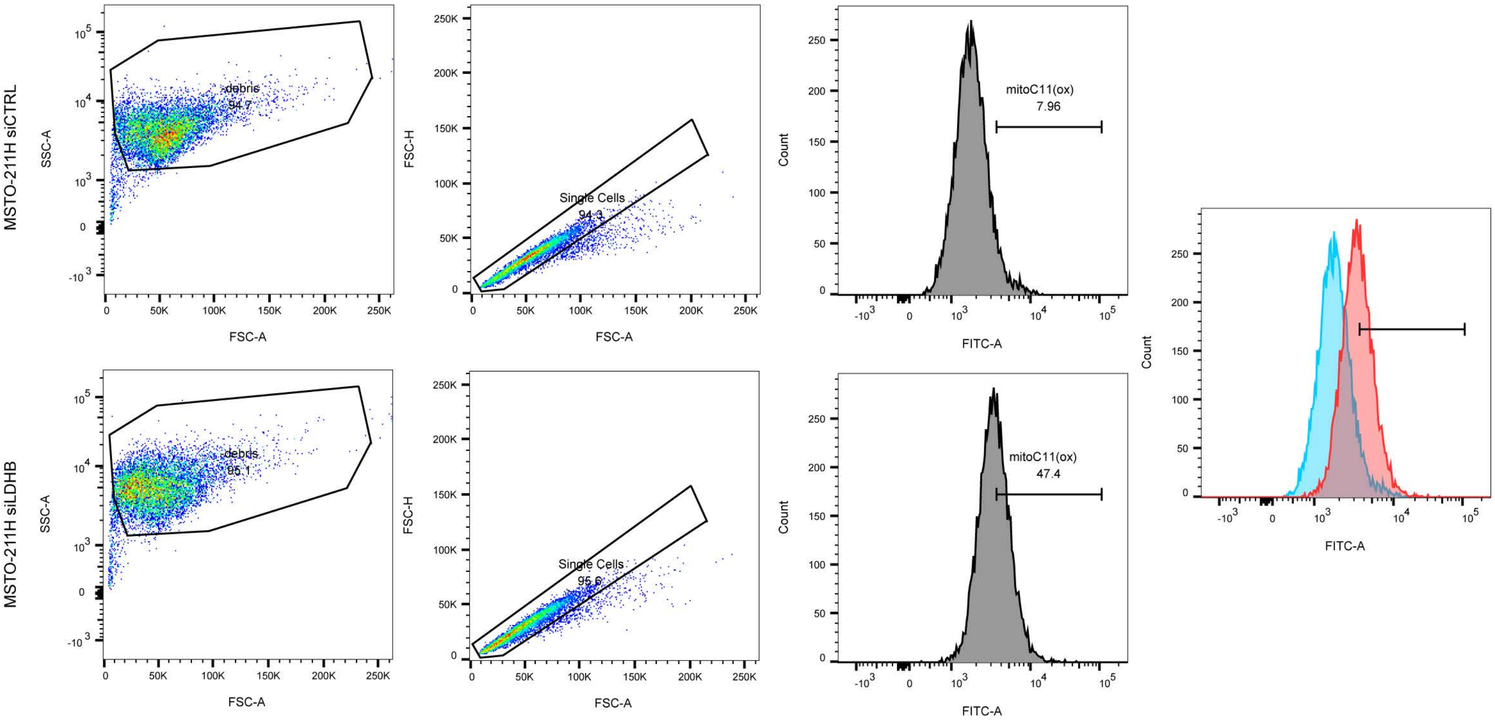

Supplementary Fig. 6 Flow cytometry gating strategy for analyzing lipid peroxidation. Representative flow cytometry gating strategy for lipid peroxidation analysis.
