# Supplementary material for: Ubiquinol-mediated suppression of mitochondria-associated ferroptosis is a targetable function of lactate dehydrogenase B in cancer
Source: Nat Commun. 2025 Mar 16;16:2597. doi: 10.1038/s41467-025-57906-3 (PMC11911438; doi:10.1038/s41467-025-57906-3)
Supplement: Supplementary file 2 — Reporting Summary [file 41467_2025_57906_MOESM2_ESM.pdf]

nature portfolio

Corresponding author(s): Ren-Wang Peng
Patrick Dorn
Thomas Michael Marti

Last updated by author(s): Dec 12, 2024

Reporting Summary

Nature Portfolio wishes to improve the reproducibility of the work that we publish. This form provides structure for consistency and transparency in reporting. For further information on Nature Portfolio policies, see our Editorial Policies and the Editorial Policy Checklist.

Statistics

For all statistical analyses, confirm that the following items are present in the figure legend, table legend, main text, or Methods section.

n/a	Confirmed	
☐	☒	The exact sample size (*n*) for each experimental group/condition, given as a discrete number and unit of measurement
☐	☒	A statement on whether measurements were taken from distinct samples or whether the same sample was measured repeatedly
☐	☒	The statistical test(s) used AND whether they are one- or two-sided<br>*Only common tests should be described solely by name; describe more complex techniques in the Methods section.*
☒	☐	A description of all covariates tested
☒	☐	A description of any assumptions or corrections, such as tests of normality and adjustment for multiple comparisons
☐	☒	A full description of the statistical parameters including central tendency (e.g. means) or other basic estimates (e.g. regression coefficient) AND variation (e.g. standard deviation) or associated estimates of uncertainty (e.g. confidence intervals)
☐	☒	For null hypothesis testing, the test statistic (e.g. *F*, *t*, *r*) with confidence intervals, effect sizes, degrees of freedom and *P* value noted<br>*Give P values as exact values whenever suitable.*
☒	☐	For Bayesian analysis, information on the choice of priors and Markov chain Monte Carlo settings
☒	☐	For hierarchical and complex designs, identification of the appropriate level for tests and full reporting of outcomes
☒	☐	Estimates of effect sizes (e.g. Cohen's *d*, Pearson's *r*), indicating how they were calculated

Our web collection on statistics for biologists contains articles on many of the points above.

Software and code

Policy information about availability of computer code

Data collection	No custom algorithms or software was used for data collection. Flow cytometry: BD FACSDiva Software v8.0.1
Data analysis	Fiji software (Fiji, RRID:SCR_002285); GraphPad Prism 9; 3D Slicer version 4.13; FlowJo v10.8.1

For manuscripts utilizing custom algorithms or software that are central to the research but not yet described in published literature, software must be made available to editors and reviewers. We strongly encourage code deposition in a community repository (e.g. GitHub). See the Nature Portfolio guidelines for submitting code & software for further information.

Data

Policy information about availability of data

All manuscripts must include a data availability statement. This statement should provide the following information, where applicable:

- Accession codes, unique identifiers, or web links for publicly available datasets
- A description of any restrictions on data availability
- For clinical datasets or third party data, please ensure that the statement adheres to our policy

All data generated during the study are available in the figures and supplementary files. For publicly available data included in this study, accession codes are provided.

Research involving human participants, their data, or biological material

Policy information about studies with human participants or human data. See also policy information about sex, gender (identity/presentation), and sexual orientation and race, ethnicity and racism.

Reporting on sex and gender	No analysis based on sex or gender was conducted.
Reporting on race, ethnicity, or other socially relevant groupings	No classification or grouping based on social variables was conducted.
Population characteristics	not applicable.
Recruitment	not applicable.
Ethics oversight	The mouse experiments were performed in accordance with animal welfare guidelines and protocols approved by the Institutional Animal Care and Ethical Committee (license number BE49_2022).

Note that full information on the approval of the study protocol must also be provided in the manuscript.

Field-specific reporting

Please select the one below that is the best fit for your research. If you are not sure, read the appropriate sections before making your selection.

[x] Life sciences [ ] Behavioural & social sciences [ ] Ecological, evolutionary & environmental sciences

For a reference copy of the document with all sections, see nature.com/documents/nr-reporting-summary-flat.pdf

Life sciences study design

All studies must disclose on these points even when the disclosure is negative.

Sample size	To detect a minimum difference of 20% in tumor volume with a significance level of 5% ($p < 0.05$) and a statistical power of 80%, a sample size calculation determined that 4 mice per group were needed. To account for potential issues, such as failed intratracheal injections or lack of tumor formation, we estimated that up to 20% of the mice might need to be excluded. As a result, each group in this study included 5 to 9 mice.
Data exclusions	No data were excluded from the analysis.
Replication	Experiments were repeated as detailed in the figure legends.
Randomization	Animals were randomly assigned to the different groups.
Blinding	The analyses were not blinded, as the same researchers conducted and evaluated the experiments.

Reporting for specific materials, systems and methods

We require information from authors about some types of materials, experimental systems and methods used in many studies. Here, indicate whether each material, system or method listed is relevant to your study. If you are not sure if a list item applies to your research, read the appropriate section before selecting a response.

Materials & experimental systems

n/a	Involved in the study	
[ ]	[x]	Antibodies
[ ]	[x]	Eukaryotic cell lines
[x]	[ ]	Palaeontology and archaeology
[ ]	[x]	Animals and other organisms
[x]	[ ]	Clinical data
[x]	[ ]	Dual use research of concern
[x]	[ ]	Plants

Methods

n/a	Involved in the study	
[ ]	[ ]	ChIP-seq
[ ]	[x]	Flow cytometry
[ ]	[ ]	MRI-based neuroimaging

Antibodies

Antibodies used	LDHB (1:10000, R&D Systems MAB9205-100), LDHA (1:1000, Cell Signaling Technology 2012S), GPX4 (1:1000, Abcam ab125066), DHODH (1: 1000, Cell Signaling Technology 26381S), FSP1 (1:1000, Proteintech 20886-1-AP), SLC7A11 (1:1000, Cell Signaling Technology 12691S), beta-actin (1:5000, Cell Signaling Technology 3700S). Secondary antibodies anti-mouse IgG (1:5000; LI-COR

	Biosciences 926-68020), anti-rabbit IgG (1:5000; LI-COR Biosciences 926-32211)
Validation	alidation for commercially available antibodies can be found using the links below:<br>LDHB https://www.rndsystems.com/products/human-lactate-dehydrogenase-b-ldhb-antibody-2057d_mab9205<br>LDHA https://www.cellsignal.com/products/primary-antibodies/ldha-antibody/2012<br>GPX4 https://www.abcam.com/products/primary-antibodies/glutathione-peroxidase-4-antibody-epncir144-ab125066.html<br>DHODH https://www.cellsignal.com/products/primary-antibodies/dhodh-e9x8r-rabbit-mab/26381<br>FSP1 https://www.ptglab.com/products/AIFM2-Antibody-20886-1-AP.htm<br>xCT/SLC7A11 https://www.cellsignal.com/products/primary-antibodies/xct-slc7a11-d2m7a-rabbit-mab/12691<br>beta-actin https://www.cellsignal.com/products/primary-antibodies/b-actin-8h10d10-mouse-mab/3700<br>Donkey anti-Mouse IgG https://www.licor.com/bio/reagents/irdye-680lt-donkey-anti-mouse-igg-secondary-antibody<br>Donkey anti-Rabbit IgG https://www.licor.com/bio/reagents/irdye-680lt-donkey-anti-rabbit-igg-secondary-antibody

Eukaryotic cell lines

Policy information about cell lines and Sex and Gender in Research

Cell line source(s)	ATCC: A549, NCI-H358, NCI-H460, NCI-H2228, NCI-H1299, H520, NCI-H2009, DMS114, SU.86.86, SKBR3, LS174T<br>RIKEN BioResource Research Center: LU99,<br>DSMZ-German Collection of Microorganisms and Cell Cultures Gmbh: PANC-1, MSTO-211H, HT1080,<br>The primary culture PF139 established from a pleural effusion of a male lung adenocarcinoma patient was generated by Balazs Hegedüs as described before (PMID: 34369083 PMCID: PMC8422071 DOI: 10.15252/emmm.202013193)
Authentication	All cell lines were authenticated by the cell line authentication service of Microsynth
Mycoplasma contamination	All cell lines were tested negative for mycoplasma contamination.
Commonly misidentified lines<br>(See ICLAC register)	None

Animals and other research organisms

Policy information about studies involving animals; ARRIVE guidelines recommended for reporting animal research, and Sex and Gender in Research

Laboratory animals	Short name, NSG: NOD.Cg-Prkdc<scid> Il2rg<tm1Wjl>/SzJ<br>Short name, Ldhb;Kras;Tp53: B6N-Ldhbtm1b(KOMP)Wtsi; B6-129S4/SvJae-Kras<tm4Tyj>/J; B6.129P2/OlaHsd-Trp53<tm1Brn><br>Short name, Rag2 x gc-/-: B10;B6-Rag2<tm1Fwa< Il2rg<tm1Wjl>
Wild animals	not applicable
Reporting on sex	Mice were randomly assigned to an experimental group regardless of their sex.
Field-collected samples	not applicable
Ethics oversight	The mouse experiments were performed in accordance with animal welfare guidelines and protocols approved by the Institutional Animal Care and Ethical Committee (license number BE49_2022).

Note that full information on the approval of the study protocol must also be provided in the manuscript.

Plants

Seed stocks	*Report on the source of all seed stocks or other plant material used. If applicable, state the seed stock centre and catalogue number. If plant specimens were collected from the field, describe the collection location, date and sampling procedures.*
Novel plant genotypes	*Describe the methods by which all novel plant genotypes were produced. This includes those generated by transgenic approaches, gene editing, chemical/radiation-based mutagenesis and hybridization. For transgenic lines, describe the transformation method, the number of independent lines analyzed and the generation upon which experiments were performed. For gene-edited lines, describe the editor used, the endogenous sequence targeted for editing, the targeting guide RNA sequence (if applicable) and how the editor was applied.*
Authentication	*Describe any authentication procedures for each seed stock used or novel genotype generated. Describe any experiments used to assess the effect of a mutation and, where applicable, how potential secondary effects (e.g. second site T-DNA insertions, mosiacism, off-target gene editing) were examined.*

ChIP-seq

Data deposition

- [ ] Confirm that both raw and final processed data have been deposited in a public database such as GEO.
- [ ] Confirm that you have deposited or provided access to graph files (e.g. BED files) for the called peaks.

Data access links<br>*May remain private before publication.*	*For "Initial submission" or "Revised version" documents, provide reviewer access links. For your "Final submission" document, provide a link to the deposited data.*
Files in database submission	*Provide a list of all files available in the database submission.*
Genome browser session<br>(e.g. UCSC)	*Provide a link to an anonymized genome browser session for "Initial submission" and "Revised version" documents only, to enable peer review. Write "no longer applicable" for "Final submission" documents.*

Methodology

Replicates	*Describe the experimental replicates, specifying number, type and replicate agreement.*
Sequencing depth	*Describe the sequencing depth for each experiment, providing the total number of reads, uniquely mapped reads, length of reads and whether they were paired- or single-end.*
Antibodies	*Describe the antibodies used for the ChIP-seq experiments; as applicable, provide supplier name, catalog number, clone name, and lot number.*
Peak calling parameters	*Specify the command line program and parameters used for read mapping and peak calling, including the ChIP, control and index files used.*
Data quality	*Describe the methods used to ensure data quality in full detail, including how many peaks are at FDR 5% and above 5-fold enrichment.*
Software	*Describe the software used to collect and analyze the ChIP-seq data. For custom code that has been deposited into a community repository, provide accession details.*

Flow Cytometry

Plots

Confirm that:

- [x] The axis labels state the marker and fluorochrome used (e.g. CD4-FITC).
- [x] The axis scales are clearly visible. Include numbers along axes only for bottom left plot of group (a 'group' is an analysis of identical markers).
- [x] All plots are contour plots with outliers or pseudocolor plots.
- [x] A numerical value for number of cells or percentage (with statistics) is provided.

Methodology

Sample preparation	Cells were harvested with TrypLE™ Select Enzyme and resuspended in 200 µL PBS containing 5 µM BODIPY™ 581/591 C11 (Invitrogen D3861) or 5 µM MitoPerOx (Abcam ab146820) for 30 minutes at 37°C to measure lipid peroxidation or mitochondrial lipid peroxidation. To measure cell death, both floating cells and adherent cells were harvested and stained with 50 µg/ml PI (Sigma-Aldrich P486) immediately before flow cytometric analysis.
Instrument	BD LSR II Flow Cytometer
Software	BD FACSDiva Software v8.0.1 was used to collect data and FlowJo_V10.8.1 was used to analyze data.
Cell population abundance	At least 10 000 cells were analyzed.
Gating strategy	The first gate was used to exclude debris based on SSC-A and FSC-A plot, then the single cell was gated based on FSC-A and FSC-H diagrams. A figure exemplifying the gating strategy was partially shown in the main figures and supplementary figures. A full gating strategy can be provided upon request.

- [ ] Tick this box to confirm that a figure exemplifying the gating strategy is provided in the Supplementary Information.

Magnetic resonance imaging

Experimental design

Design type — *Indicate task or resting state; event-related or block design.*

Design specifications — *Specify the number of blocks, trials or experimental units per session and/or subject, and specify the length of each trial or block (if trials are blocked) and interval between trials.*

Behavioral performance measures — *State number and/or type of variables recorded (e.g. correct button press, response time) and what statistics were used to establish that the subjects were performing the task as expected (e.g. mean, range, and/or standard deviation across subjects).*

Acquisition

Imaging type(s) — *Specify: functional, structural, diffusion, perfusion.*

Field strength — *Specify in Tesla*

Sequence & imaging parameters — *Specify the pulse sequence type (gradient echo, spin echo, etc.), imaging type (EPI, spiral, etc.), field of view, matrix size, slice thickness, orientation and TE/TR/flip angle.*

Area of acquisition — *State whether a whole brain scan was used OR define the area of acquisition, describing how the region was determined.*

Diffusion MRI ☐ Used ☐ Not used

Preprocessing

Preprocessing software — *Provide detail on software version and revision number and on specific parameters (model/functions, brain extraction, segmentation, smoothing kernel size, etc.).*

Normalization — *If data were normalized/standardized, describe the approach(es): specify linear or non-linear and define image types used for transformation OR indicate that data were not normalized and explain rationale for lack of normalization.*

Normalization template — *Describe the template used for normalization/transformation, specifying subject space or group standardized space (e.g. original Talairach, MNI305, ICBM152) OR indicate that the data were not normalized.*

Noise and artifact removal — *Describe your procedure(s) for artifact and structured noise removal, specifying motion parameters, tissue signals and physiological signals (heart rate, respiration).*

Volume censoring — *Define your software and/or method and criteria for volume censoring, and state the extent of such censoring.*

Statistical modeling & inference

Model type and settings — *Specify type (mass univariate, multivariate, RSA, predictive, etc.) and describe essential details of the model at the first and second levels (e.g. fixed, random or mixed effects; drift or auto-correlation).*

Effect(s) tested — *Define precise effect in terms of the task or stimulus conditions instead of psychological concepts and indicate whether ANOVA or factorial designs were used.*

Specify type of analysis: ☐ Whole brain ☐ ROI-based ☐ Both

Statistic type for inference — *Specify voxel-wise or cluster-wise and report all relevant parameters for cluster-wise methods.*

(See Eklund et al. 2016)

Correction — *Describe the type of correction and how it is obtained for multiple comparisons (e.g. FWE, FDR, permutation or Monte Carlo).*

Models & analysis

n/a	Involved in the study
☐	☐ Functional and/or effective connectivity
☐	☐ Graph analysis
☐	☐ Multivariate modeling or predictive analysis

Functional and/or effective connectivity	*Report the measures of dependence used and the model details (e.g. Pearson correlation, partial correlation, mutual information).*
Graph analysis	*Report the dependent variable and connectivity measure, specifying weighted graph or binarized graph, subject- or group-level, and the global and/or node summaries used (e.g. clustering coefficient, efficiency, etc.).*
Multivariate modeling and predictive analysis	*Specify independent variables, features extraction and dimension reduction, model, training and evaluation metrics.*
