# Supplementary figures and images for: Ubiquinol-mediated suppression of mitochondria-associated ferroptosis is a targetable function of lactate dehydrogenase B in cancer
Source: Nat Commun. 2025 Mar 16;16:2597. doi: 10.1038/s41467-025-57906-3 (PMC11911438; doi:10.1038/s41467-025-57906-3)

Fig.3a

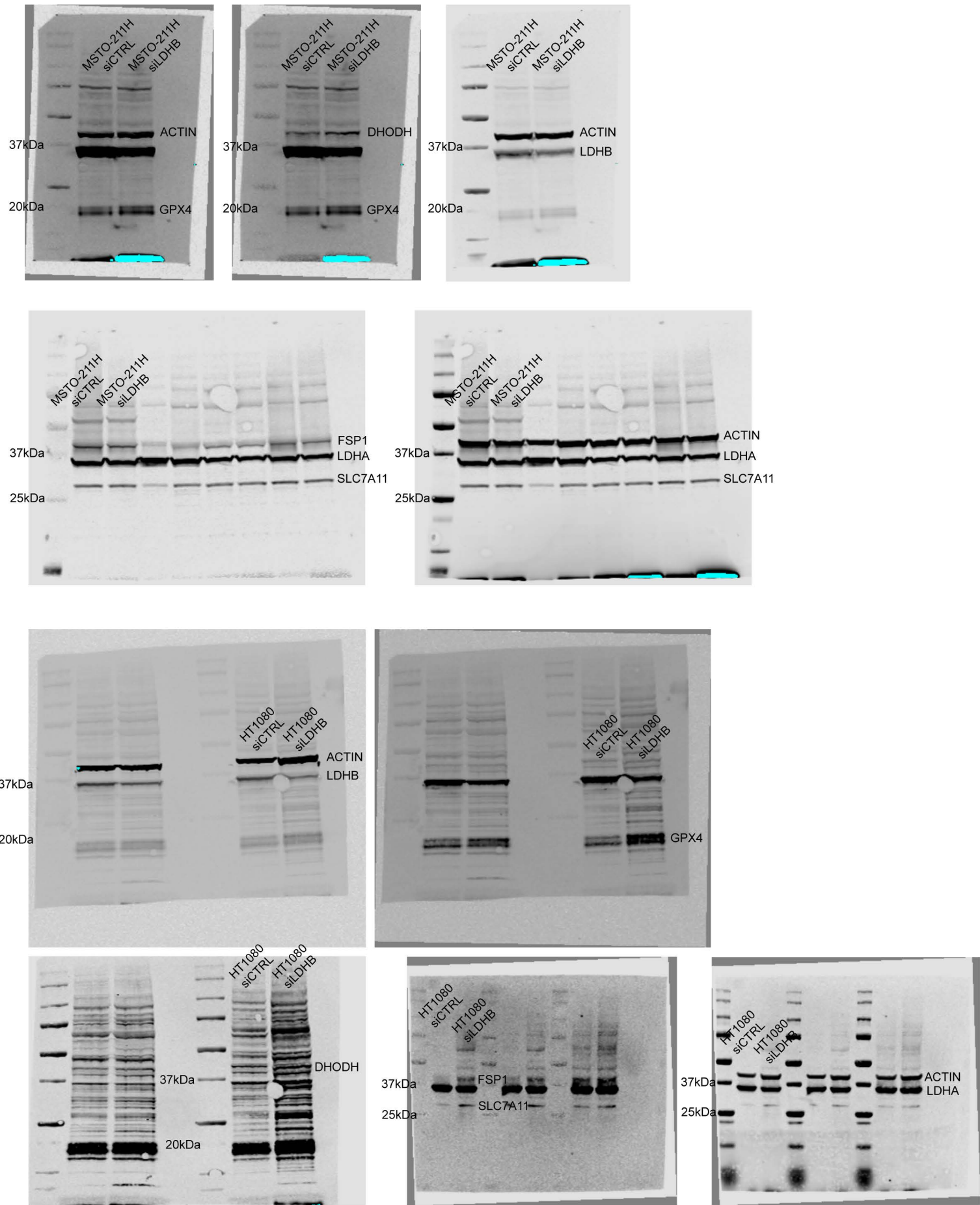

Fig.3a

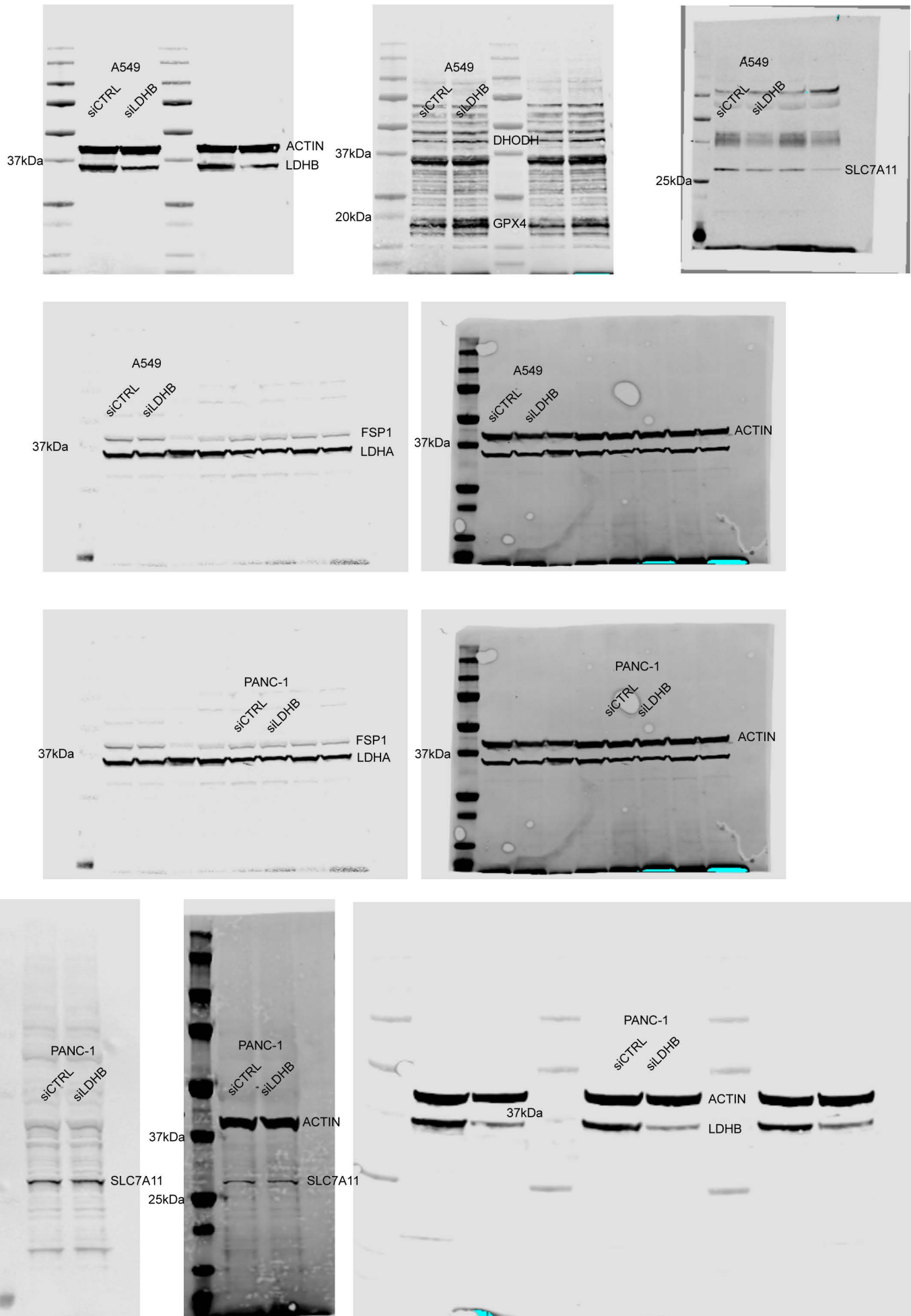

Fig.3a

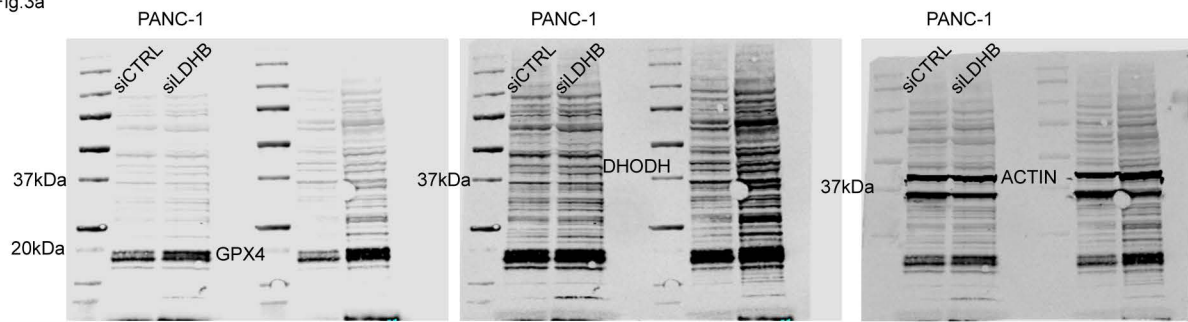

Fig.3e

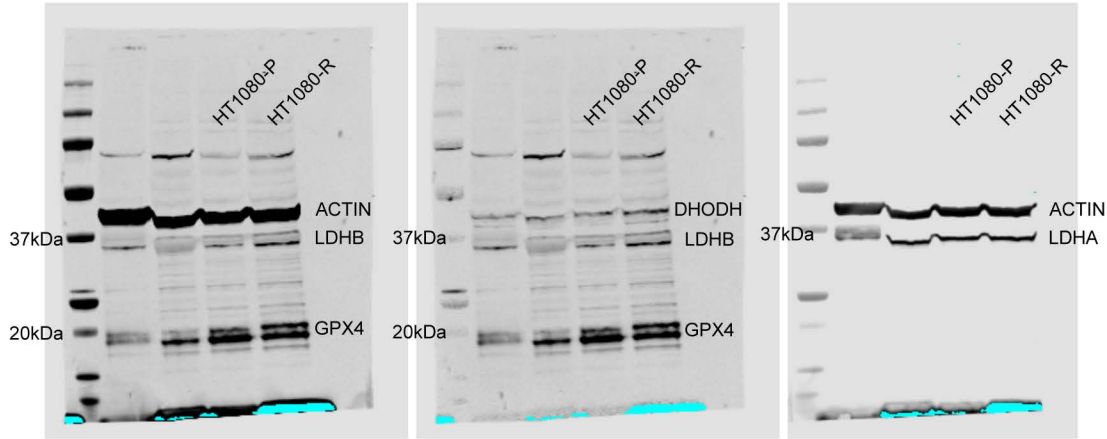

Fig.S2g

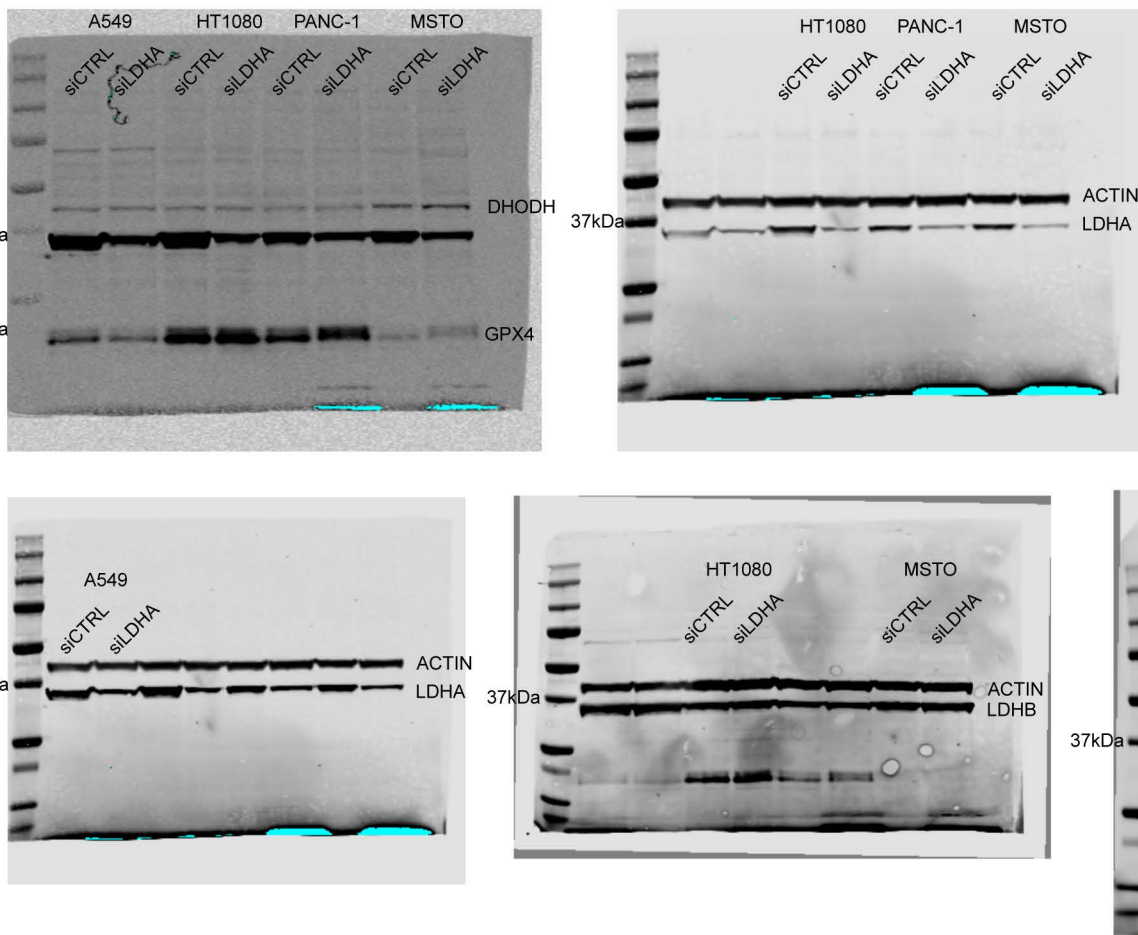

Fig.S3d

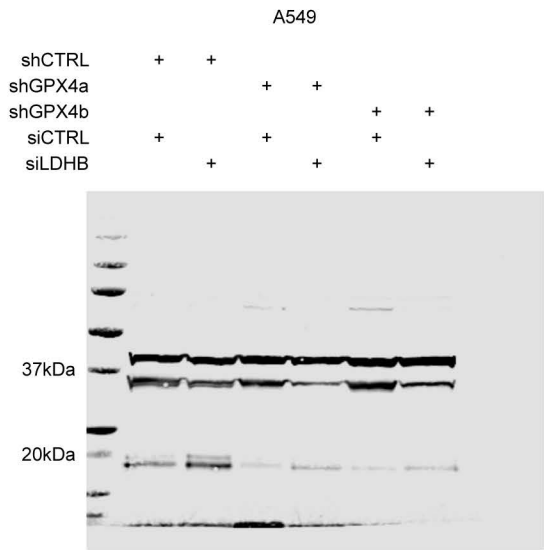

Fig.S3e

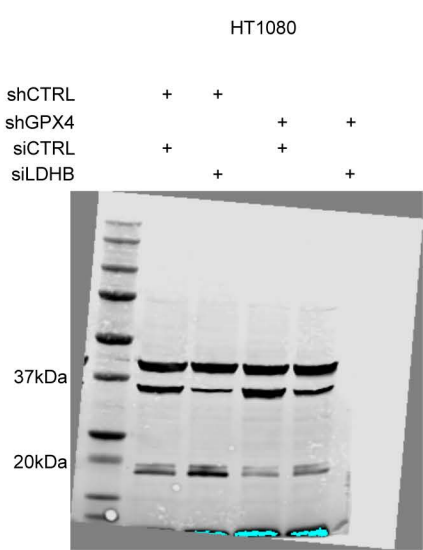

Fig.S4e

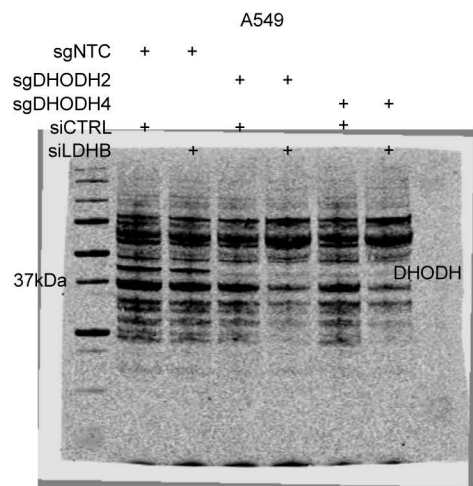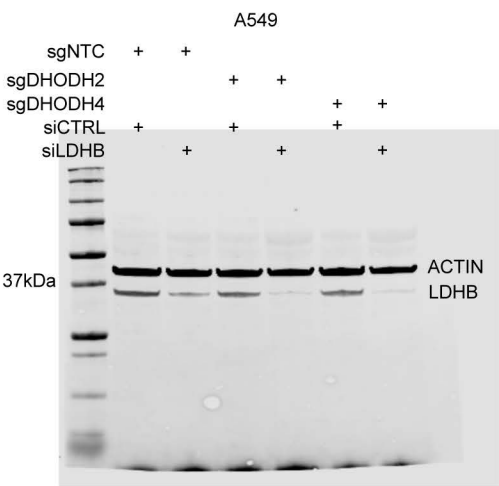

Fig.S4f

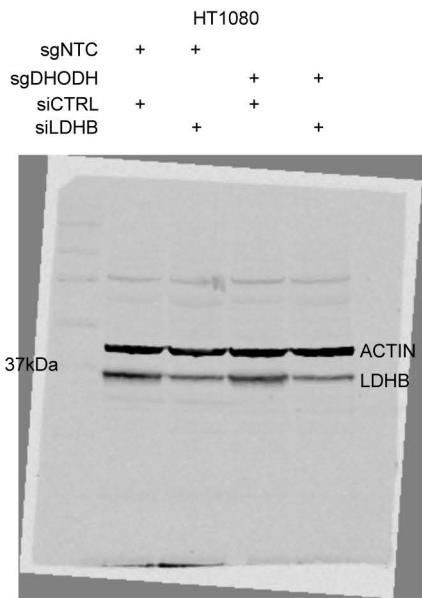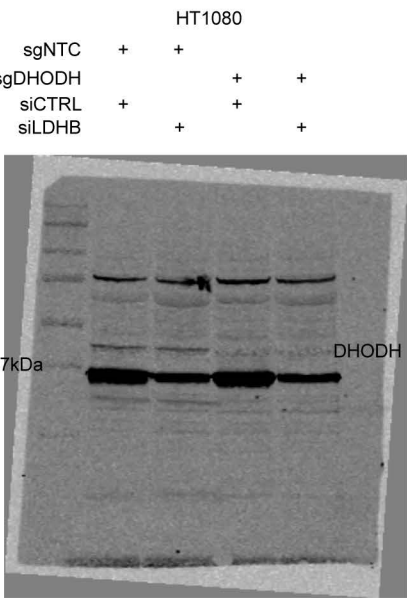

Fig.S4h

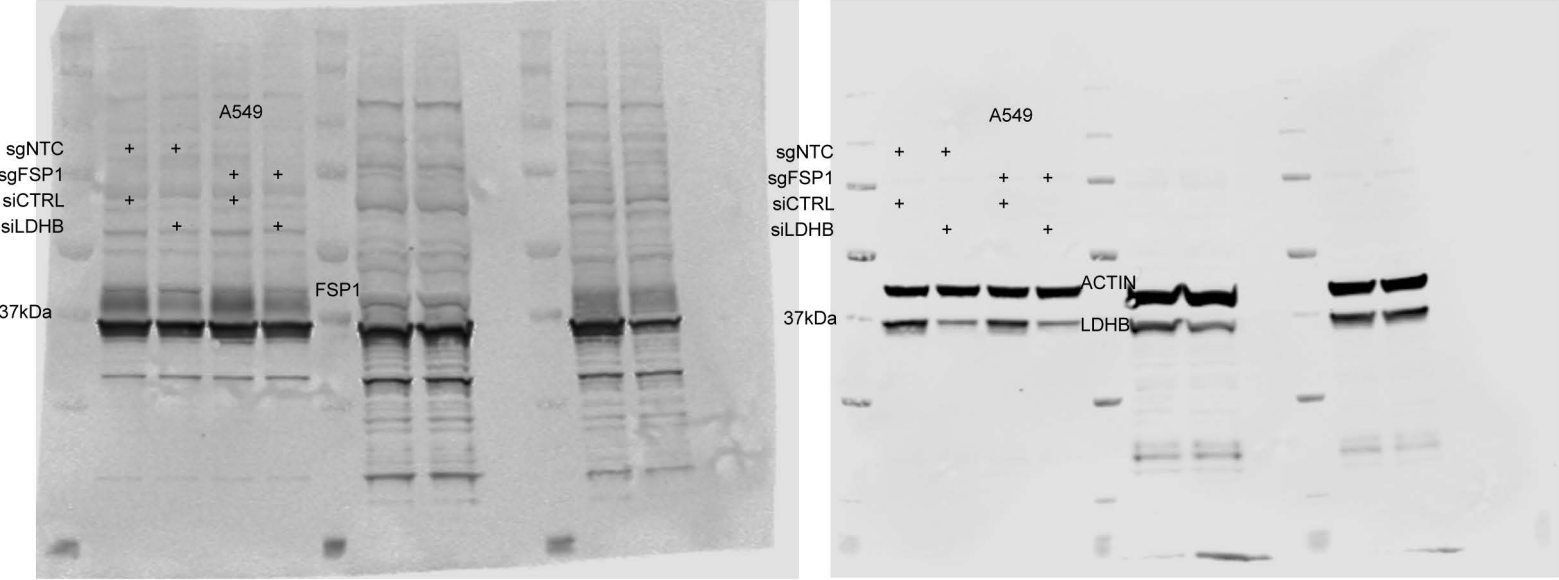

Fig.S4i

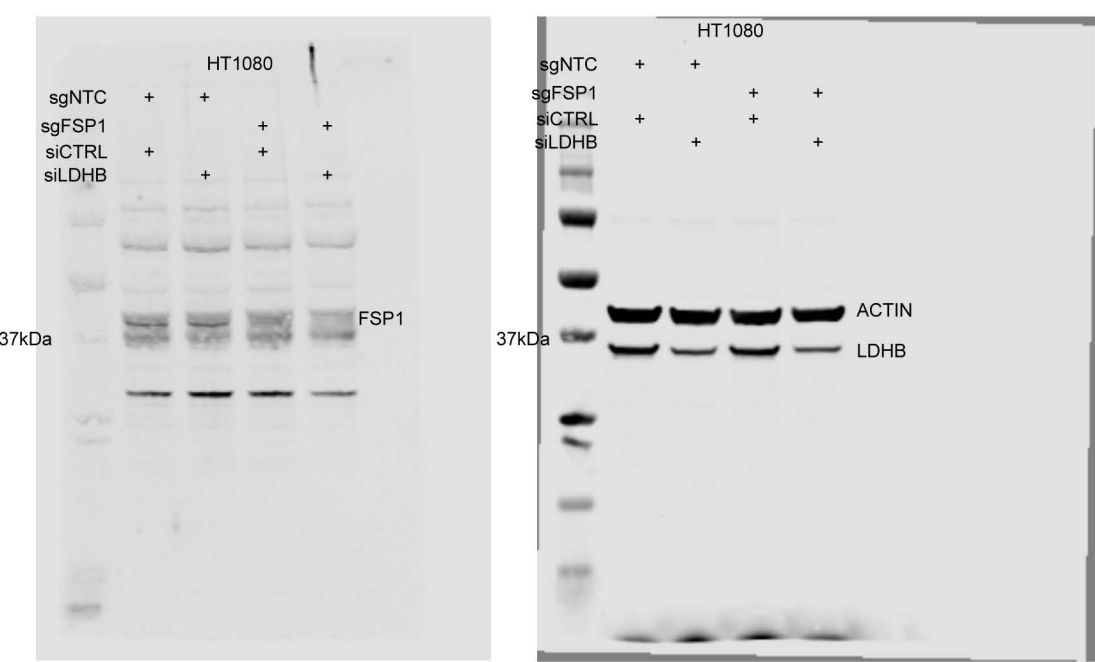

Fig.S4w

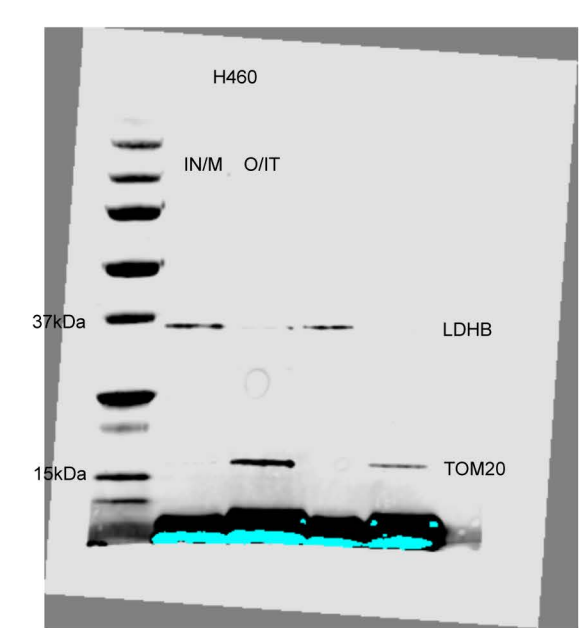

Supplement: Supplementary file 4 — Source Data [file 41467_2025_57906_MOESM4_ESM.zip › Source data/Raw WB scan.pdf]
